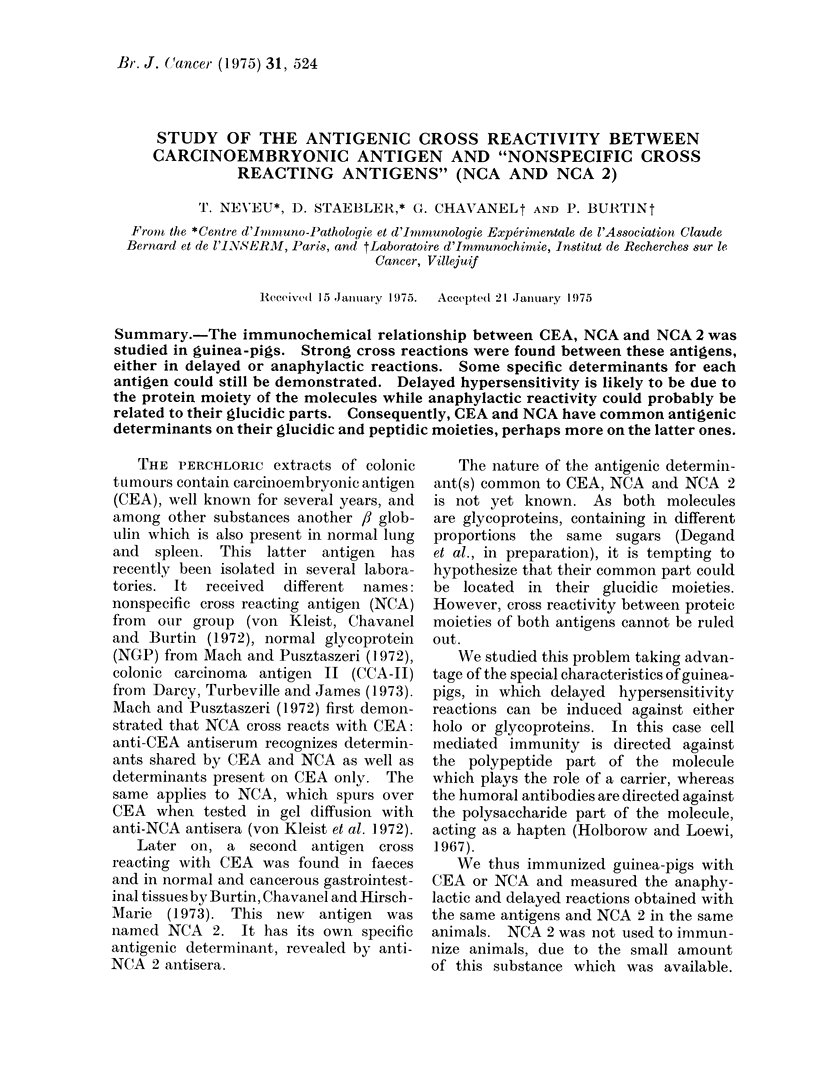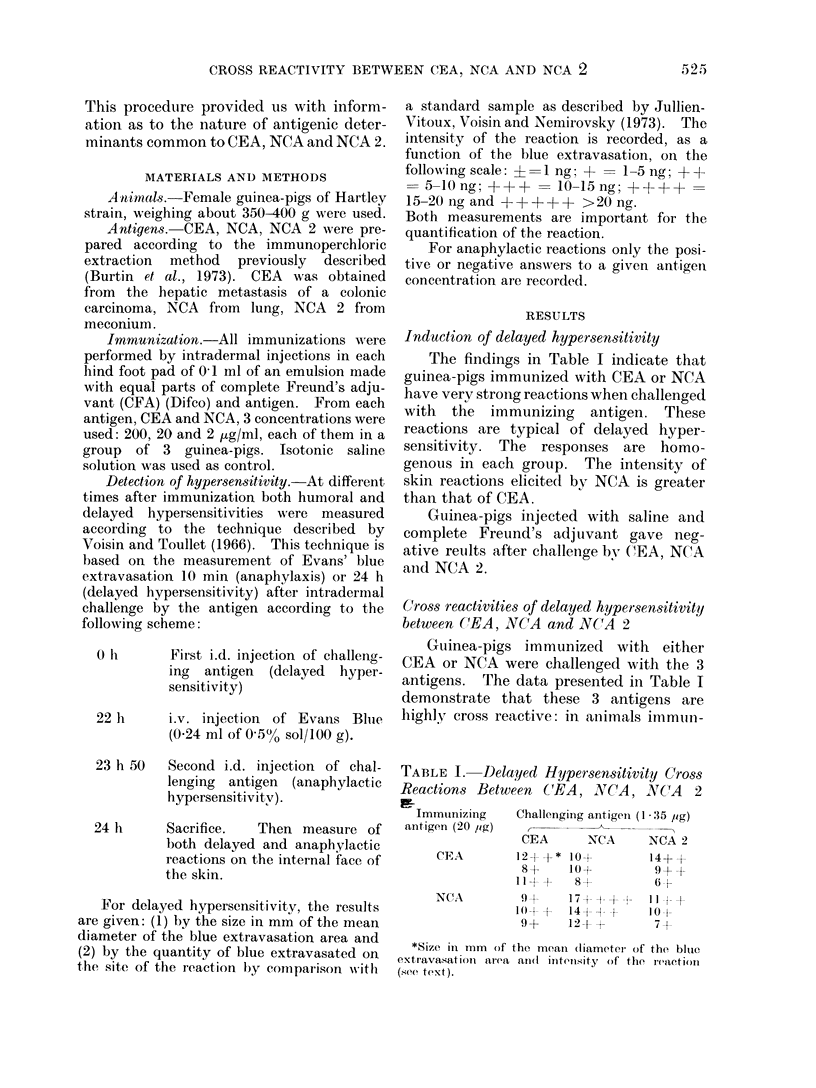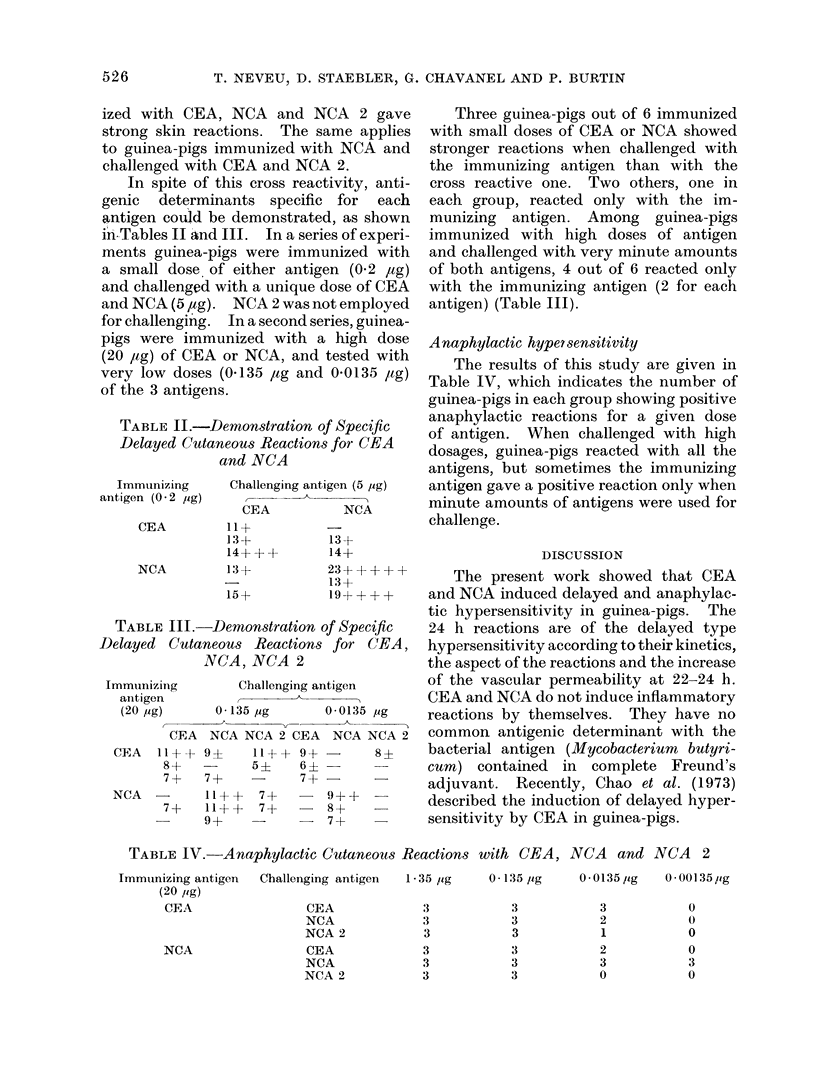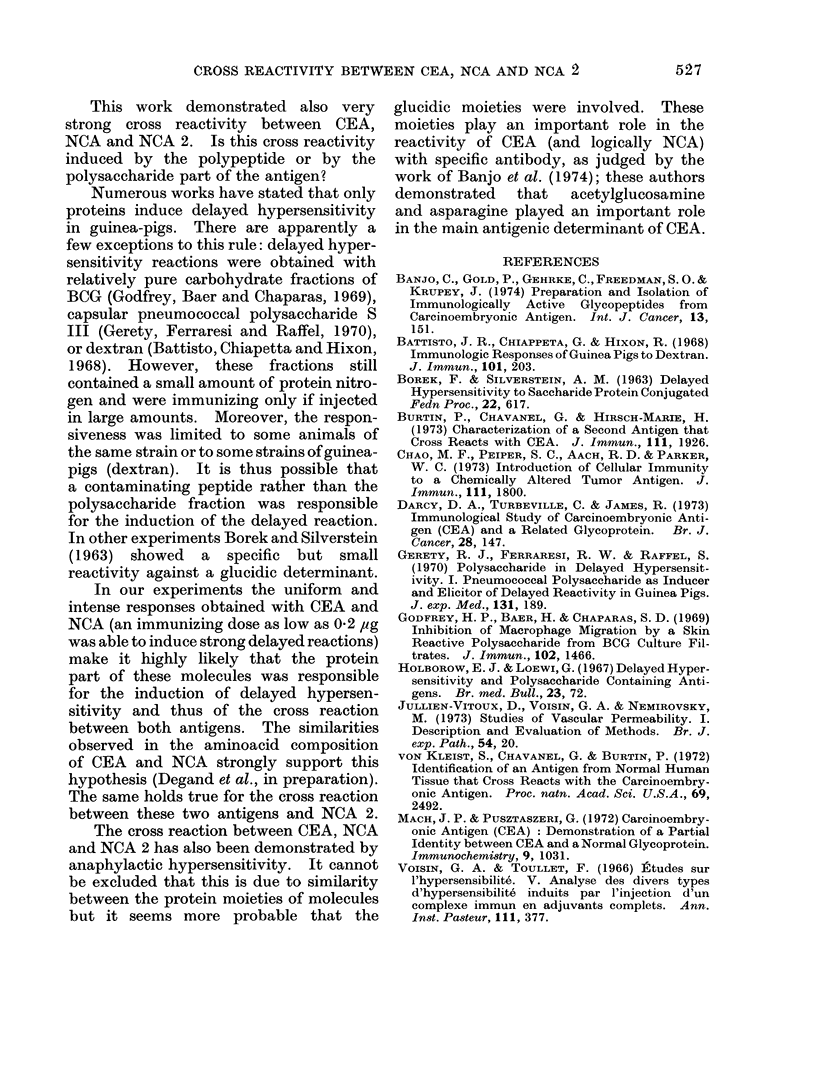# Study of the antigenic cross reactivity between carcinoembryonic antigen and "nonspecific cross reacting antigens" (NCA and NCA 2).

**DOI:** 10.1038/bjc.1975.92

**Published:** 1975-05

**Authors:** T. Neveu, D. Staebler, G. Chavanel, P. Burtin

## Abstract

The immunochemical relationship between CEA, NCA and NCA 2 was studied in guinea-pigs. Strong cross reactions were found between these antigens, either in delayed or anaphylactic reactions. Some specific determinants for each antigen could still be demonstrated. Delayed hypersensitivity is likely to be due to the protein moiety of the molecules while anaphylactic reactivity could probably be related to their glucidic parts. Consequently, CEA and NCA have common antigenic determinants on their glucidic and peptidic moieties, perhaps more on the latter ones.


Br. J. Cancer (1975) **31**, 524

### STUDY OF THE ANTIGENIC CROSS REACTIVITY BETWEEN CARCINOEMBRYONIC ANTIGEN AND "NONSPECIFIC CROSS REACTING ANTIGENS" (NCA AND NCA 2)

T. NEVEU*, D. STAEBLER,* G. CHAVANEL† AND P. BURTIN†

*From the *Centre d'Immuno-Pathologie et d'Immunologie Expérimentale de l'Association Claude Bernard et de l'INSERM, Paris, and †Laboratoire d'Immunochimie, Institut de Recherches sur le Cancer, Villejuif*

Received 15 January 1975. Accepted 21 January 1975

**Summary.—The immunochemical relationship between CEA, NCA and NCA 2 was studied in guinea-pigs. Strong cross reactions were found between these antigens, either in delayed or anaphylactic reactions. Some specific determinants for each antigen could still be demonstrated. Delayed hypersensitivity is likely to be due to the protein moiety of the molecules while anaphylactic reactivity could probably be related to their glucidic parts. Consequently, CEA and NCA have common antigenic determinants on their glucidic and peptidic moieties, perhaps more on the latter ones.**

THE PERCHLORIC extracts of colonic tumours contain carcinoembryonic antigen (CEA), well known for several years, and among other substances another $\beta$ globulin which is also present in normal lung and spleen. This latter antigen has recently been isolated in several laboratories. It received different names: nonspecific cross reacting antigen (NCA) from our group (von Kleist, Chavanel and Burtin (1972), normal glycoprotein (NGP) from Mach and Pusztaszeri (1972), colonic carcinoma antigen II (CCA-II) from Darcy, Turbeville and James (1973). Mach and Pusztaszeri (1972) first demonstrated that NCA cross reacts with CEA: anti-CEA antiserum recognizes determinants shared by CEA and NCA as well as determinants present on CEA only. The same applies to NCA, which spurs over CEA when tested in gel diffusion with anti-NCA antisera (von Kleist *et al.* 1972).

Later on, a second antigen cross reacting with CEA was found in faeces and in normal and cancerous gastrointestinal tissues by Burtin, Chavanel and Hirsch-Marie (1973). This new antigen was named NCA 2. It has its own specific antigenic determinant, revealed by anti-NCA 2 antisera.

The nature of the antigenic determinant(s) common to CEA, NCA and NCA 2 is not yet known. As both molecules are glycoproteins, containing in different proportions the same sugars (Degand *et al.*, in preparation), it is tempting to hypothesize that their common part could be located in their glucidic moieties. However, cross reactivity between proteic moieties of both antigens cannot be ruled out.

We studied this problem taking advantage of the special characteristics of guinea-pigs, in which delayed hypersensitivity reactions can be induced against either holo or glycoproteins. In this case cell mediated immunity is directed against the polypeptide part of the molecule which plays the role of a carrier, whereas the humoral antibodies are directed against the polysaccharide part of the molecule, acting as a hapten (Holborow and Loewi, 1967).

We thus immunized guinea-pigs with CEA or NCA and measured the anaphylactic and delayed reactions obtained with the same antigens and NCA 2 in the same animals. NCA 2 was not used to immunize animals, due to the small amount of this substance which was available.

This procedure provided us with information as to the nature of antigenic determinants common to CEA, NCA and NCA 2.

### MATERIALS AND METHODS

*Animals.*—Female guinea-pigs of Hartley strain, weighing about 350–400 g were used.

*Antigens.*—CEA, NCA, NCA 2 were prepared according to the immunoperchloric extraction method previously described (Burtin *et al.*, 1973). CEA was obtained from the hepatic metastasis of a colonic carcinoma, NCA from lung, NCA 2 from meconium.

*Immunization.*—All immunizations were performed by intradermal injections in each hind foot pad of 0·1 ml of an emulsion made with equal parts of complete Freund's adjuvant (CFA) (Difco) and antigen. From each antigen, CEA and NCA, 3 concentrations were used: 200, 20 and 2 μg/ml, each of them in a group of 3 guinea-pigs. Isotonic saline solution was used as control.

*Detection of hypersensitivity.*—At different times after immunization both humoral and delayed hypersensitivities were measured according to the technique described by Voisin and Toullet (1966). This technique is based on the measurement of Evans' blue extravasation 10 min (anaphylaxis) or 24 h (delayed hypersensitivity) after intradermal challenge by the antigen according to the following scheme:

| | |
|---|---|
| 0 h | First i.d. injection of challenging antigen (delayed hypersensitivity) |
| 22 h | i.v. injection of Evans Blue (0·24 ml of 0·5% sol/100 g). |
| 23 h 50 | Second i.d. injection of challenging antigen (anaphylactic hypersensitivity). |
| 24 h | Sacrifice. Then measure of both delayed and anaphylactic reactions on the internal face of the skin. |

For delayed hypersensitivity, the results are given: (1) by the size in mm of the mean diameter of the blue extravasation area and (2) by the quantity of blue extravasated on the site of the reaction by comparison with a standard sample as described by Jullien-Vitoux, Voisin and Nemirovsky (1973). The intensity of the reaction is recorded, as a function of the blue extravasation, on the following scale: $\pm = 1$ ng; $+ = 1$–5 ng; $++ = 5$–10 ng; $+++ = 10$–15 ng; $++++ = 15$–20 ng and $+++++ > 20$ ng.
Both measurements are important for the quantification of the reaction.

For anaphylactic reactions only the positive or negative answers to a given antigen concentration are recorded.

### RESULTS

### *Induction of delayed hypersensitivity*

The findings in Table I indicate that guinea-pigs immunized with CEA or NCA have very strong reactions when challenged with the immunizing antigen. These reactions are typical of delayed hypersensitivity. The responses are homogenous in each group. The intensity of skin reactions elicited by NCA is greater than that of CEA.

Guinea-pigs injected with saline and complete Freund's adjuvant gave negative reults after challenge by CEA, NCA and NCA 2.

### *Cross reactivities of delayed hypersensitivity between CEA, NCA and NCA 2*

Guinea-pigs immunized with either CEA or NCA were challenged with the 3 antigens. The data presented in Table I demonstrate that these 3 antigens are highly cross reactive: in animals immun-

TABLE I.—*Delayed Hypersensitivity Cross Reactions Between CEA, NCA, NCA 2*

| Immunizing antigen (20 μg) | Challenging antigen (1·35 μg) | | |
|---|---|---|---|
| | CEA | NCA | NCA 2 |
| CEA | 12++* | 10+ | 14++ |
| | 8+ | 10+ | 9++ |
| | 11++ | 8+ | 6+ |
| NCA | 9+ | 17++++ | 11++ |
| | 10++ | 14+++ | 10+ |
| | 9+ | 12++ | 7+ |

*Size in mm of the mean diameter of the blue extravasation area and intensity of the reaction (see text).

ized with CEA, NCA and NCA 2 gave strong skin reactions. The same applies to guinea-pigs immunized with NCA and challenged with CEA and NCA 2.

In spite of this cross reactivity, antigenic determinants specific for each antigen could be demonstrated, as shown in Tables II and III. In a series of experiments guinea-pigs were immunized with a small dose of either antigen (0·2 μg) and challenged with a unique dose of CEA and NCA (5 μg). NCA 2 was not employed for challenging. In a second series, guinea-pigs were immunized with a high dose (20 μg) of CEA or NCA, and tested with very low doses (0·135 μg and 0·0135 μg) of the 3 antigens.

TABLE II.—*Demonstration of Specific Delayed Cutaneous Reactions for CEA and NCA*

| Immunizing antigen (0·2 μg) | Challenging antigen (5 μg) CEA | NCA |
|---|---|---|
| CEA | 11+ | — |
| | 13+ | 13+ |
| | 14+ + + | 14+ |
| NCA | 13+ | 23+ + + + + |
| | — | 13+ |
| | 15+ | 19+ + + + |

TABLE III.—*Demonstration of Specific Delayed Cutaneous Reactions for CEA, NCA, NCA 2*

| Immunizing antigen (20 μg) | Challenging antigen 0·135 μg | | | 0·0135 μg | | |
|---|---|---|---|---|---|---|
| | CEA | NCA | NCA 2 | CEA | NCA | NCA 2 |
| CEA | 11+ + | 9± | 11+ + | 9+ | — | 8± |
| | 8+ | — | 5± | 6± | — | — |
| | 7+ | 7+ | — | 7+ | — | — |
| NCA | — | 11+ + | 7+ | — | 9+ + | — |
| | 7+ | 11+ + | 7+ | — | 8+ | — |
| | — | 9+ | — | — | 7+ | — |

Three guinea-pigs out of 6 immunized with small doses of CEA or NCA showed stronger reactions when challenged with the immunizing antigen than with the cross reactive one. Two others, one in each group, reacted only with the immunizing antigen. Among guinea-pigs immunized with high doses of antigen and challenged with very minute amounts of both antigens, 4 out of 6 reacted only with the immunizing antigen (2 for each antigen) (Table III).

### *Anaphylactic hypersensitivity*

The results of this study are given in Table IV, which indicates the number of guinea-pigs in each group showing positive anaphylactic reactions for a given dose of antigen. When challenged with high dosages, guinea-pigs reacted with all the antigens, but sometimes the immunizing antigen gave a positive reaction only when minute amounts of antigens were used for challenge.

### DISCUSSION

The present work showed that CEA and NCA induced delayed and anaphylactic hypersensitivity in guinea-pigs. The 24 h reactions are of the delayed type hypersensitivity according to their kinetics, the aspect of the reactions and the increase of the vascular permeability at 22–24 h. CEA and NCA do not induce inflammatory reactions by themselves. They have no common antigenic determinant with the bacterial antigen (*Mycobacterium butyricum*) contained in complete Freund's adjuvant. Recently, Chao *et al.* (1973) described the induction of delayed hypersensitivity by CEA in guinea-pigs.

TABLE IV.—*Anaphylactic Cutaneous Reactions with CEA, NCA and NCA 2*

| Immunizing antigen (20 μg) | Challenging antigen | 1·35 μg | 0·135 μg | 0·0135 μg | 0·00135 μg |
|---|---|---|---|---|---|
| CEA | CEA | 3 | 3 | 3 | 0 |
| | NCA | 3 | 3 | 2 | 0 |
| | NCA 2 | 3 | 3 | 1 | 0 |
| NCA | CEA | 3 | 3 | 2 | 0 |
| | NCA | 3 | 3 | 3 | 3 |
| | NCA 2 | 3 | 3 | 0 | 0 |

This work demonstrated also very strong cross reactivity between CEA, NCA and NCA 2. Is this cross reactivity induced by the polypeptide or by the polysaccharide part of the antigen?

Numerous works have stated that only proteins induce delayed hypersensitivity in guinea-pigs. There are apparently a few exceptions to this rule: delayed hypersensitivity reactions were obtained with relatively pure carbohydrate fractions of BCG (Godfrey, Baer and Chaparas, 1969), capsular pneumococcal polysaccharide S III (Gerety, Ferraresi and Raffel, 1970), or dextran (Battisto, Chiapetta and Hixon, 1968). However, these fractions still contained a small amount of protein nitrogen and were immunizing only if injected in large amounts. Moreover, the responsiveness was limited to some animals of the same strain or to some strains of guinea-pigs (dextran). It is thus possible that a contaminating peptide rather than the polysaccharide fraction was responsible for the induction of the delayed reaction. In other experiments Borek and Silverstein (1963) showed a specific but small reactivity against a glucidic determinant.

In our experiments the uniform and intense responses obtained with CEA and NCA (an immunizing dose as low as 0·2 $\mu$g was able to induce strong delayed reactions) make it highly likely that the protein part of these molecules was responsible for the induction of delayed hypersensitivity and thus of the cross reaction between both antigens. The similarities observed in the aminoacid composition of CEA and NCA strongly support this hypothesis (Degand *et al.*, in preparation). The same holds true for the cross reaction between these two antigens and NCA 2.

The cross reaction between CEA, NCA and NCA 2 has also been demonstrated by anaphylactic hypersensitivity. It cannot be excluded that this is due to similarity between the protein moieties of molecules but it seems more probable that the glucidic moieties were involved. These moieties play an important role in the reactivity of CEA (and logically NCA) with specific antibody, as judged by the work of Banjo *et al.* (1974); these authors demonstrated that acetylglucosamine and asparagine played an important role in the main antigenic determinant of CEA.

### REFERENCES

Banjo, C., Gold, P., Gehrke, C., Freedman, S. O. & Krupey, J. (1974) Preparation and Isolation of Immunologically Active Glycopeptides from Carcinoembryonic Antigen. *Int. J. Cancer*, **13**, 151.

Battisto, J. R., Chiappeta, G. & Hixon, R. (1968) Immunologic Responses of Guinea Pigs to Dextran. *J. Immun.*, **101**, 203.

Borek, F. & Silverstein, A. M. (1963) Delayed Hypersensitivity to Saccharide Protein Conjugated *Fedn Proc.*, **22**, 617.

Burtin, P., Chavanel, G. & Hirsch-Marie, H. (1973) Characterization of a Second Antigen that Cross Reacts with CEA. *J. Immun.*, **111**, 1926.

Chao, M. F., Peiper, S. C., Aach, R. D. & Parker, W. C. (1973) Introduction of Cellular Immunity to a Chemically Altered Tumor Antigen. *J. Immun.*, **111**, 1800.

Darcy, D. A., Turbeville, C. & James, R. (1973) Immunological Study of Carcinoembryonic Antigen (CEA) and a Related Glycoprotein. *Br. J. Cancer*, **28**, 147.

Gerety, R. J., Ferraresi, R. W. & Raffel, S. (1970) Polysaccharide in Delayed Hypersensitivity. I. Pneumococcal Polysaccharide as Inducer and Elicitor of Delayed Reactivity in Guinea Pigs. *J. exp. Med.*, **131**, 189.

Godfrey, H. P., Baer, H. & Chaparas, S. D. (1969) Inhibition of Macrophage Migration by a Skin Reactive Polysaccharide from BCG Culture Filtrates. *J. Immun.*, **102**, 1466.

Holborow, E. J. & Loewi, G. (1967) Delayed Hypersensitivity and Polysaccharide Containing Antigens. *Br. med. Bull.*, **23**, 72.

Jullien-Vitoux, D., Voisin, G. A. & Nemirovsky, M. (1973) Studies of Vascular Permeability. I. Description and Evaluation of Methods. *Br. J. exp. Path.*, **54**, 20.

von Kleist, S., Chavanel, G. & Burtin, P. (1972) Identification of an Antigen from Normal Human Tissue that Cross Reacts with the Carcinoembryonic Antigen. *Proc. natn. Acad. Sci. U.S.A.*, **69**, 2492.

Mach, J. P. & Pusztaszeri, G. (1972) Carcinoembryonic Antigen (CEA) : Demonstration of a Partial Identity between CEA and a Normal Glycoprotein. *Immunochemistry*, **9**, 1031.

Voisin, G. A. & Toullet, F. (1966) Études sur l'hypersensibilité. V. Analyse des divers types d'hypersensibilité induits par l'injection d'un complexe immun en adjuvants complets. *Ann. Inst. Pasteur*, **111**, 377.